# Supplementary material for: Thin-Film-Engineered Self-Assembly of 3D Coaxial Microfluidics with a Tunable Polyimide Membrane for Bioelectronic Power
Source: Nanomicro Lett. 2026 Apr 22;18:342. doi: 10.1007/s40820-026-02188-7 (PMC13103006; doi:10.1007/s40820-026-02188-7)
Supplement: Supplementary file 6 — Supplementary file6 (DOCX 5938 kb) [file 40820_2026_2188_MOESM6_ESM.docx]

Supporting Information for

**Thin-Film-Engineered Self-Assembly of 3D Coaxial Microfluidics with a Tunable Polyimide Membrane for Bioelectronic Power**

Aleksandr I. Egunov^1^*, Hongmei Tang^1^, Pablo E. Saenz^1^, Dmitriy D. Karnaushenko^1^, Yumin Luo^1,2^, Chao Zhong^3^, Xinyu Wang^3^, Yang Huang^4^, Pavel Fedorov^1^, Leandro Merces^1,5^, Minshen Zhu^1^*, Daniil Karnaushenko^1^* and Oliver G. Schmidt^1,6,7^*

^1^ Research Center for Materials, Architectures and Integration of Nanomembranes, Chemnitz University of Technology, Chemnitz, Germany

^2^ Chair of Microsystems Technology, Ruhr University Bochum, Bochum, Germany

^3^ Shenzhen Key Laboratory of Materials Synthetic Biology, Key Laboratory of Quantitative Synthetic Biology, Shenzhen Institute of Synthetic Biology, Shenzhen Institutes of Advanced Technology, Chinese Academy of Sciences, Shenzhen, P. R. China

^4^ Departament of Physics and Materials Science, City Univercity of Hong Kong, Hong Kong, P. R. China

^5^ Ilum School of Science, Brazilian Center for Research in Energy and Materials, Campinas, Brazil

^6^ Material Systems for Nanoelectronics, Chemnitz University of Technology, Chemnitz, Germany

^7^ Nanophysics, Dresden University of Technology, Dresden, Germany

*Corresponding authors. E-mail: [aleksandr.egunov@main.tu-chemnitz.de](mailto:aleksandr.egunov@main.tu-chemnitz.de) (Aleksandr I. Egunov); [minshen.zhu@main.tu-chemnitz.de](mailto:minshen.zhu@main.tu-chemnitz.de) (Minshen Zhu); [daniil.karnaushenko@main.tu-chemnitz.de](mailto:daniil.karnaushenko@main.tu-chemnitz.de) (Daniil Karnaushenko); [oliver.schmidt@main.tu-chemnitz.de](mailto:oliver.schmidt@main.tu-chemnitz.de) (Oliver G. Schmidt)

**Supplementary Information**

**Extended data for Fig. 3, Fig. S6 and Fig. S7**

The observed impedance and capacitance behavior (Fig. S6) can be directly explained by the physics of electric double-layer (EDL) formation. At low ionic strength (10^-5^–10^-3^ M KCl), the Debye screening length (*λ_D_*) (Supplementary Table S1) is extended, reaching ~100 nm at 10⁻⁵ M and ~10 nm at 10^-3^ M. In this regime, counterion accumulation at the electrode is incomplete, resulting in low double-layer capacitance and high impedance. This effect is particularly pronounced in 2D IDEs, where limited effective surface area and larger inter-electrode distances restrict efficient charge compensation.

At intermediate concentrations (10^-2^–10^-1^ M), *λ_D_* contracts to ~3–1 nm, enabling more effective EDL charging and a marked increase in capacitance. In this range, the coaxial geometry of 3D IDEs provides a decisive advantage: the increased interfacial area and shortened ionic pathways reduce impedance significantly compared to 2D IDEs. PPEs, by contrast, offer extended planar interfaces that sustain strong and uniform EDL formation, leading to the lowest observed impedance across the frequency range.

At high ionic strength (1 M KCl), *λ_D_* falls below 1 nm, producing saturated double-layer capacitance at all electrode types. Here, impedance is dominated by bulk electrolyte conductivity, and the performance gap between electrode architectures diminishes. Nonetheless, PPEs maintain the most stable capacitive response, while 3D IDEs preserve broader frequency-dependent charge storage capacity.

These findings confirm that coaxial self-rolled electrodes provide favorable conditions for EDL formation across a broad concentration range, combining low impedance with stable capacitive behavior. Such characteristics are critical for microbial fuel cell operation, where efficient charge transfer and minimized resistive losses directly translate into improved bioelectrochemical performance.

**Ionic strength dependence (KCl solutions, Fig. 3c and Fig. S6).**

At 1 M KCl, PPEs exhibited impedance values as low as ~1.5 × 10^3^ Ω at 100 Hz, compared to ~1×10^5^ Ω for 2D IDEs and ~1 × 10⁴ Ω for 3D IDEs. At lower concentration (10^-5^ M KCl), impedance increased to ~5×10^6^ Ω for 2D IDEs, ~1 × 10⁶ Ω for 3D IDEs, and ~3×10^5^ Ω for PPEs.

This reduction can be described by the classical Debye screening relation:

$$\boldsymbol{\lambda}_{\boldsymbol{D}}\boldsymbol{=}\sqrt{\frac{\boldsymbol{\varepsilon}\boldsymbol{k}_{\boldsymbol{B}}\boldsymbol{T}}{\boldsymbol{2}\boldsymbol{N}_{\boldsymbol{A}}\boldsymbol{e}^{\boldsymbol{2}}\boldsymbol{I}}}$$

where *λ_D_* is the Debye length, *ε* is the dielectric permittivity of water (ε ≈ 80),

*k_B_* is Boltzmann constant = 1.380×10^-23^ J·K⁻¹ , *T* — Absolute temperature, assumed 298 K (25 °C), *N_A_* — Avogadro’s number = 6.022×10^23^ mol⁻¹, *I* — Ionic strength (mol·L⁻¹), defined as

$$\boldsymbol{I=}\frac{\boldsymbol{1}}{\boldsymbol{2}}\sum\boldsymbol{C}_{\boldsymbol{i}}\boldsymbol{Z}_{\boldsymbol{i}}^{\boldsymbol{2}}$$

where *C_i_* ​ is molar concentration of ion i, and *Z_i_* its valence.

Increasing *I* from 10^-5^ M to 1 M reduces *λ_D_* from ~100 nm to ~0.3 nm, explaining the improved charge screening and lowered impedance. Capacitance estimates from the Z′–C′ plots confirm this: PPEs reach ~3×10^-9^ F at 1 M KCl, compared to ~2×10^-10^ F for 2D IDEs under the same conditions.

Dielectric dependence (ethanol–water mixtures, Fig. S7).

At fixed ionic content, decreasing the solvent dielectric constant from ε ≈ 80 (pure water) to *ε* ≈ 25 (pure ethanol) systematically increased impedance. For PPEs, |Z| at 100 Hz rose from ~1×10^4^ Ω (water) to ~1×10^7^ Ω (ethanol), i.e. a three-order-of-magnitude shift.

The capacitance followed the expected scaling:

$$\boldsymbol{C\sim}\frac{\boldsymbol{\varepsilon}}{\boldsymbol{\lambda}_{\boldsymbol{D}}}$$

where *λ_D_* remains constant at fixed ionic concentration. This results in a direct proportionality of capacitance with ε. Experimentally, PPE capacitance decreased from ~2×10^-9^ F (water) to ~7×10^-11^ F (ethanol).

2D IDEs displayed smaller changes (~1×10^-10^ F to ~5×10^-11^ F) because of their strongly localized field lines, while 3D IDEs showed intermediate sensitivity.

**Extended data for Fig. S8**

Proton exchange in alkali-treated polyimide membranes

The alkali (NaOH) treatment of PI PEM partially hydrolyzes the imide rings of polyimide (PI) into their open-chain poly(amic acid) form. This transformation introduces carboxylate (–COO⁻) and amide (–CONH–) groups that enhance ionic transport and water affinity, beneficial for proton conduction. The reaction proceeds via nucleophilic attack of hydroxide ions on the imide carbonyl carbon, followed by ring-opening to yield amic acid intermediates.

In the FTIR spectra (Fig. S9a), pristine PI shows characteristic imide peaks at:

1775 cm⁻¹ (C=O asymmetric stretch)

1720 cm⁻¹ (C=O symmetric stretch)

1380 cm⁻¹ (C–N stretch)

720 cm⁻¹ (imide ring deformation)

After NaOH exposure, new and shifted features appear:

Broad O–H/N–H stretching (3200–3600 cm⁻¹): from hydroxyl and amide groups in poly(amic acid).

Weakened imide C=O peaks (1775 and 1720 cm⁻¹): indicating ring cleavage.

New amide C=O band (~1650 cm⁻¹): characteristic of amic acid formation.

New COO⁻ symmetric stretching (~1420 cm⁻¹): confirming partial conversion to carboxylate.

The improvement in ionic transport after alkali treatment is consistent with hydrolysis of imide groups in PI:

–CO–NH–CO–  →    –COOH + –CONH– or  –COO^-^NA^+^ .

The repeat unit of the PI contains two imide linkages per monomer, which are the primary sites for hydroxide attack. Controlled NaOH hydrolysis converts a fraction of imide rings into poly(amic)-like structures bearing amide and carboxylate/carboxylic groups. In FTIR this is manifested by a reduced and broadened imide C=O envelope (~1775 and 1720 cm⁻¹), a shoulder/increase around ~1650 cm⁻¹ (amide C=O / amic acid), increased O–H/N–H absorption (~3400 cm⁻¹), and modest growth near 1420 cm⁻¹ (COO⁻ symmetric). Excessive hydrolysis produces a high density of hydrophilic groups, causing strong swelling, gelation and partial dissolution in aqueous media, a condition we avoid by selecting an intermediate target resistance (~250 Ω at 10³ Hz).

Electrochemical impedance spectroscopy (EIS) was used to quantify membrane resistance as a function of alkali treatment time. The total impedance is represented as

$$\boldsymbol{Z}\left( \boldsymbol{\omega} \right)\boldsymbol{\approx}\boldsymbol{Z}^{\boldsymbol{'}}\left( \boldsymbol{\omega} \right)\boldsymbol{+j}\boldsymbol{Z}^{\boldsymbol{'}}\boldsymbol{'}\left( \boldsymbol{\omega} \right)$$

where $\boldsymbol{Z}^{\boldsymbol{'}}\left( \boldsymbol{\omega} \right)$ is the real component (resistive contribution) and $\boldsymbol{Z}^{\boldsymbol{'}}\boldsymbol{'}\left( \boldsymbol{\omega} \right)$ is the imaginary component (capacitive contribution). For thin membranes, the dominant parameter is the real resistance at mid frequencies (10²–10⁴ Hz), where electrode polarization is negligible and bulk ionic conduction dominates.

The membrane resistance was extracted from the Bode plot at 10³ Hz,

$$\boldsymbol{R}_{\boldsymbol{mem}}\boldsymbol{\approx Z'(\omega=}\boldsymbol{10}^{\boldsymbol{3}}\boldsymbol{Hz)}$$

giving values of ~10⁶ Ω for pristine PI, ~10⁵ Ω after 10² min of NaOH treatment, and ~10³ Ω after 10³ min (Fig. S9b). At very long treatment times (>7.5 × 10³ min), *R_mem_* increased again due to membrane fragilisation and partial loss of structural integrity.

While Nafion™ 117 exhibited the lowest resistance (~10² Ω), treated PI reached comparable performance at optimal treatment times (10³–5x10^3^ min), with a significant mechanical advantage in multilayer coaxial integration. In contrast, Kapton™ remained insulating with *R_mem_*>10^7^ Ω

Thus, alkali-treated PI provides a tunable balance between ionic conductivity and structural robustness, making it particularly suited for Swiss-roll µMFC architectures where membrane winding introduces regions of reduced effective thickness.

**Supplementary Tables and Figures**

**Table S1** Calculated Debye screening length (*λ_D_*) for KCl solutions

| KCl Concentration (M) | Debye Length, *λ_D_* (nm) |
| --- | --- |
| 10^-5^ M | 96.3 |
| 10^-4^ M | 30.5 |
| 10^-3^ M | 9.63 |
| 10^-2^ M | 3.05 |
| 10^-1^ M | 0.96 |
| 1 M | 0.3 |

**Table S2** Architectural and functional comparison of membrane-based sub-1 mL µMFCs

| Ref. | Anolyte volume (A.v.), nL. | Total volume (T.v.), nL. | Architecture/Fabrication | Membrane/Separator | Maximum volumetric power density normalized per A.v., mW/cm^3^ | Maximum volumetric  power density normalized per T.v., mW/cm^3^ | Key features/ Functional notes |
| --- | --- | --- | --- | --- | --- | --- | --- |
| [16] | 1500 | 5500 | Vertically stacked dual-chamber, microfluidic | Nafion™ 117 | 0.015 | 0.004 | Early on-chip µMFC demonstration. |
| [47] | 100000 | 200000 | Planar, glass slide assembly, gasket-defined | Nafion™ 117 | 3.3 | 1.65 | Focus on characteristic length; planar chambers. |
| [32] | 4500 | 9000 | Planar MEMS, PDMS-defined chambers | CMI 7000 | 2.3 | 1.15 | High power density; uses conductive Geobacter biofilm; high coulombic efficiency. |
| [33] | 5000 | n.a. | Planar, two-compartment flow cell, serpentine channels | Nafion™ 117 | 0.9 | n.a | Continuous flow operation; serpentine channel design; carbon paper electrodes. |
| [48] | 58800 | n.a. | Microfluidic flow-through with 3D graphene foam anode | Nafion™ 117 | 0.745 | n.a. | Flow-through anode chamber; 3D graphene foam for intimate microbe interaction;  fast mass transport. |
| [49] | 1250 | n.a. | Planar, Si-based with vertical CNT anode | Nafion™ 117 | 0.392 | n.a. | High surface-area vertically aligned carbon nanotubes anode; compact total anolyte volume  (1.25 µL). |
| [50] | 10400 | 20800 | Planar MEMS, silicon double-chamber | Nafion™ 117 | 0.315 and 0.33 | 0.156 and 0.165 | Focus on anode surface modification (thiol SAMs) to reduce start-up time. |
| This work | 550 | 800 | **Coaxial Swiss-roll, strain-driven self-assembly** | **Tunable Polyimide** | **3.1** | 2.1 | **Coaxial channels; integrated & tunable PEM; dual-mode operation; monolithic 3D integration.** |


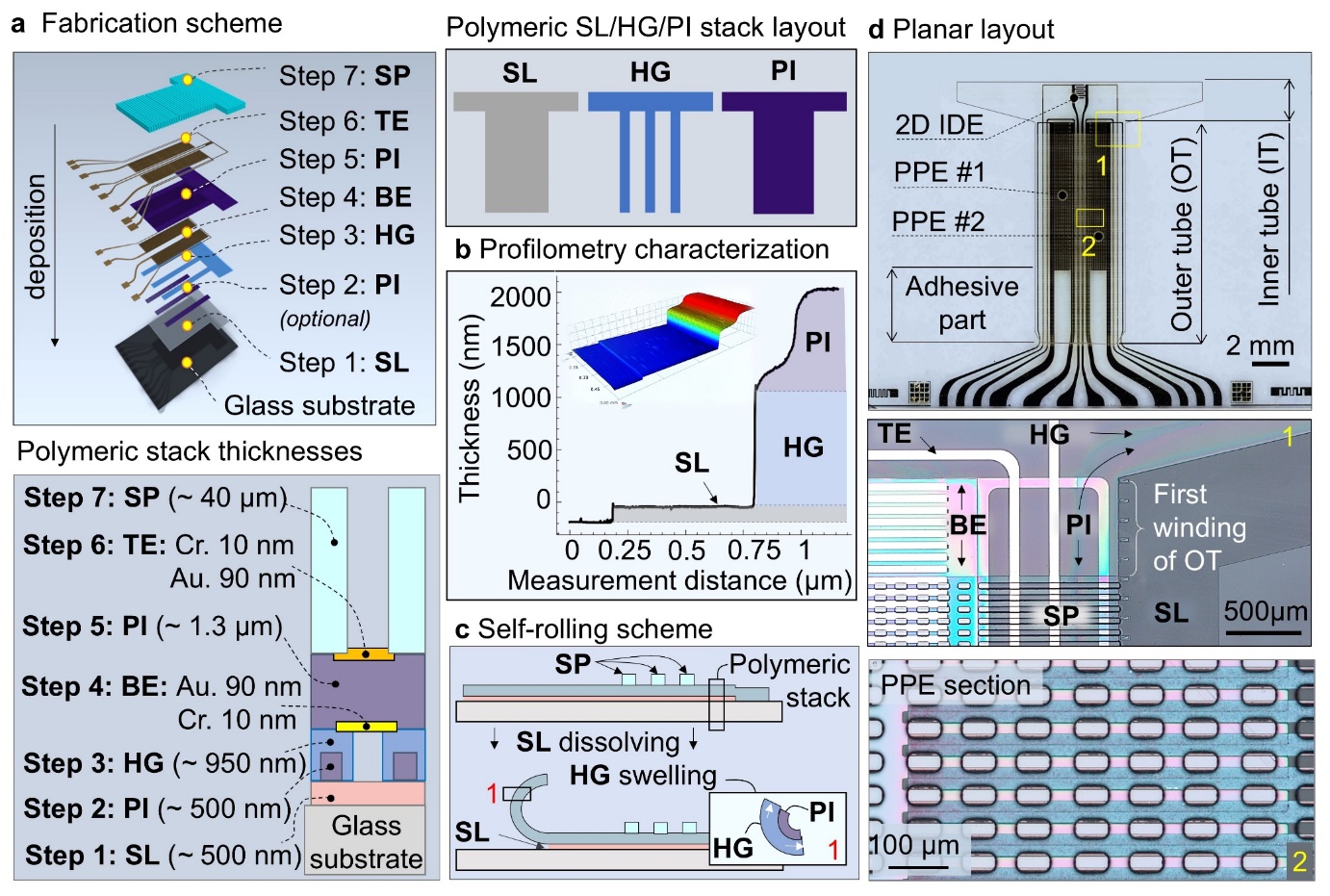


**Fig. S1** Fabrication process and structural layout of the coaxial Swiss-roll device. a Sequential thin-film deposition on a glass substrate, including sacrificial layer (SL), polyimide (PI), hydrogel (HG), bottom electrode (BE), top electrode (TE), and structural polymer (SP). Low panel: Cross-sectional schematic of the polymer stack with representative thicknesses: SL (~500 nm), PI (~500 nm, optional), HG (~950 nm), BE (Cr 10 nm / Au 90 nm), PI (~1.3 µm), TE (Cr 10 nm / Au 90 nm), and SP (~40 µm). Scale bars: 100 µm, 2 mm, and 500 µm. Right panel: schematic layout of polymeric SL/HG/PI stack layers. b Profilometry characterization with 3D map of SL/HG/PI polymeric stack. c Self-rolling mechanism: selective dissolution of the SL combined with HG swelling induces strain mismatch within the multilayer stack, resulting in autonomous tubular formation. d Planar device layout prior to rolling, showing the 2D interdigitated electrode (IDE), PPE sections, adhesive region, and the defined outer tube (OT) and inner tube (IT).


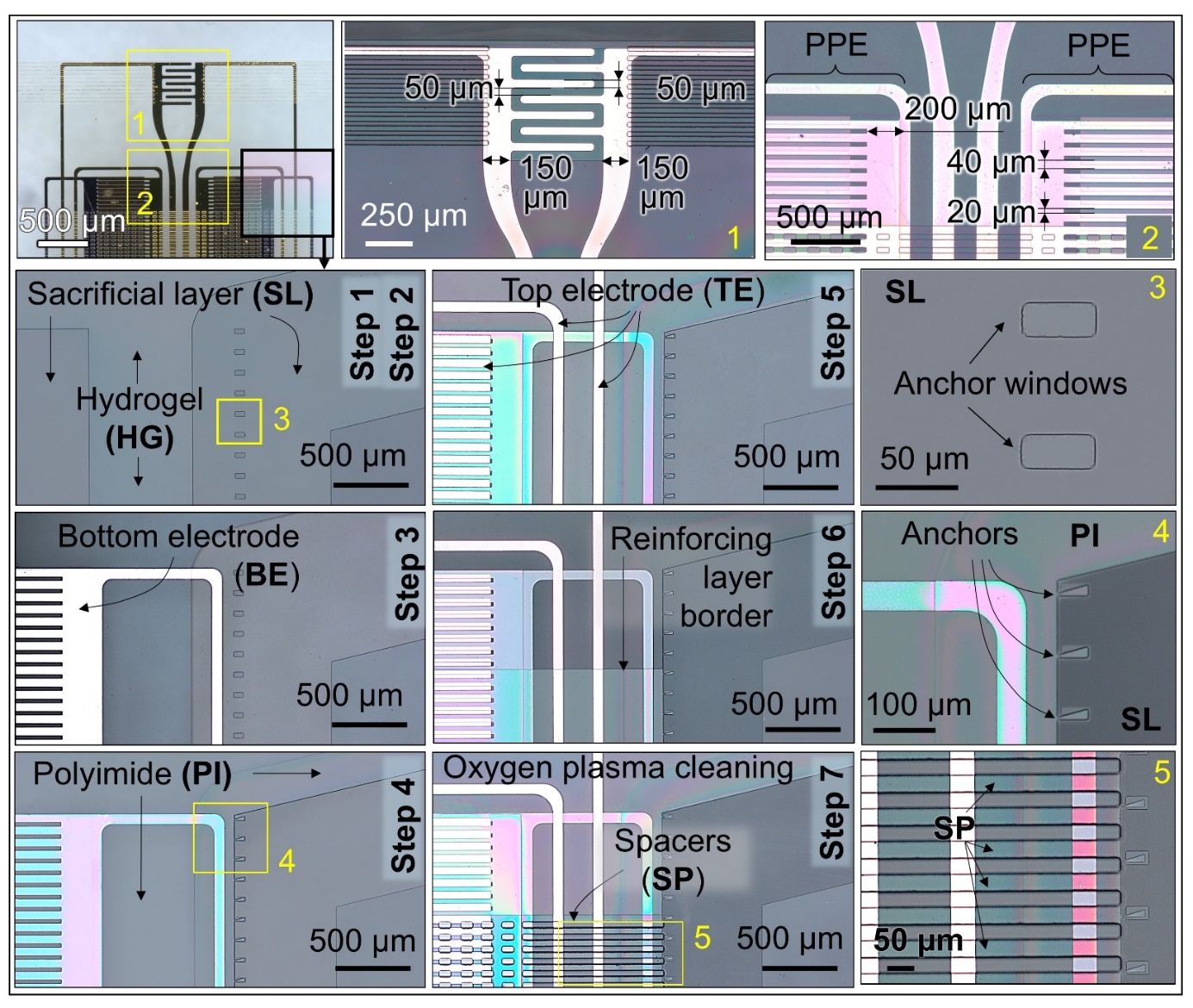


**Fig. S2** Detailed microfabrication sequence. Step-by-step photolithographic process for the polymeric nanomembrane stack: (Step 1) Sacrificial layer (SL) patterning. (Step 2) Hydrogel (HG) deposition and patterning. (Step 3) Sputtering and patterning of the bottom electrode (BE, Cr/Au). (Step 4) Spin-coating and patterning of the first polyimide (PI) layer. (Step 5) Sputtering and patterning of the top electrode (TE, Cr/Au). (Step 6) Deposition and patterning of a thicker PI reinforcing layer for mechanical stability. (Step 7) Oxygen plasma cleaning and final deposition/patterning of SU-8 spacer (SP) bars. Insets 1, 2: Detailed dimensions of the electrode structures. Insets 3, 4: Design and integration of lithographic anchors to prevent lateral rolling. Inset 5: Close-up view of the SU-8 spacer bars.


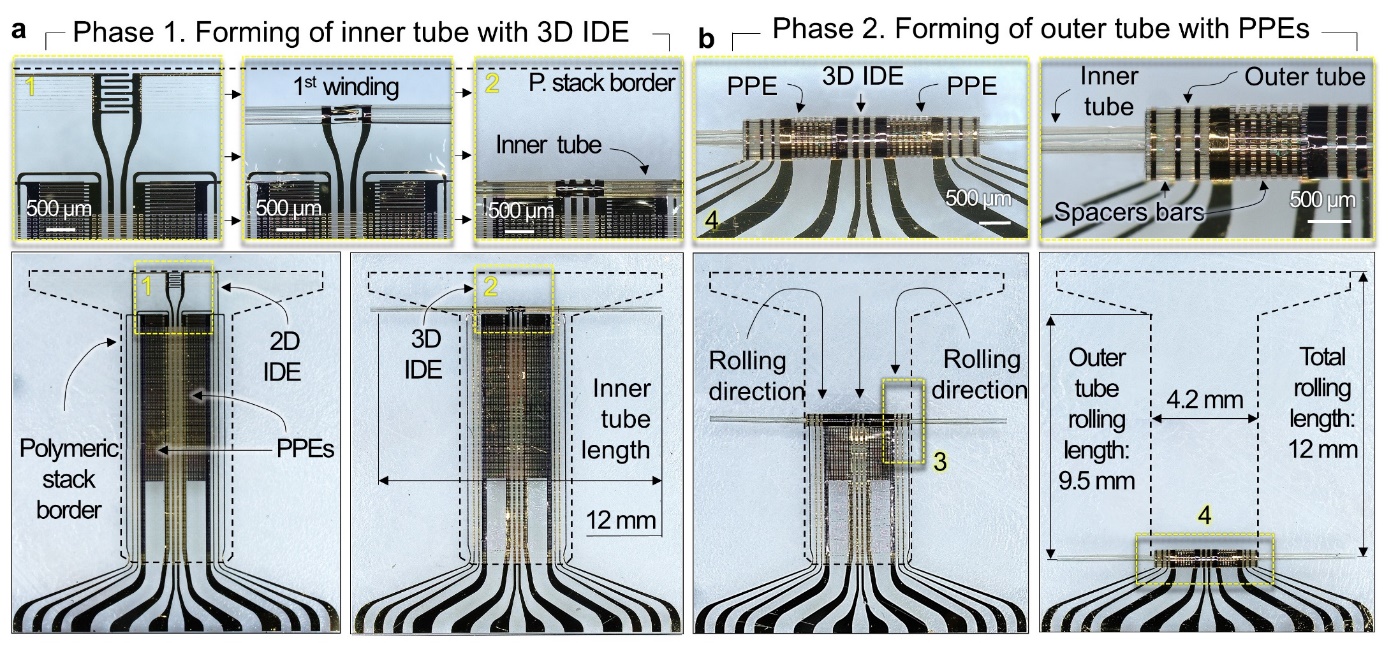


**Fig. S3** Sequential self-assembly of the 3D coaxial architecture. The strain-driven rolling process occurs in two distinct phases, guided by lithographic anchors and a polyimide (PI) reinforcing layer to ensure the correct sequence. **a** Phase 1: Inner tube formation. Overview image and corresponding zoomed views (Insets 1, 2) showing the initial rolling stage that transforms the 2D interdigitated electrodes (IDEs) into the 3D inner tube. **b** Phase 2: Outer tube formation. Overview image and corresponding zoomed views (Insets 3, 4) showing the subsequent rolling stage, where the outer structure encases the inner tube to form the complete coaxial architecture with integrated parallel-plate electrodes (PPEs). The final device has a total length of 12 mm.


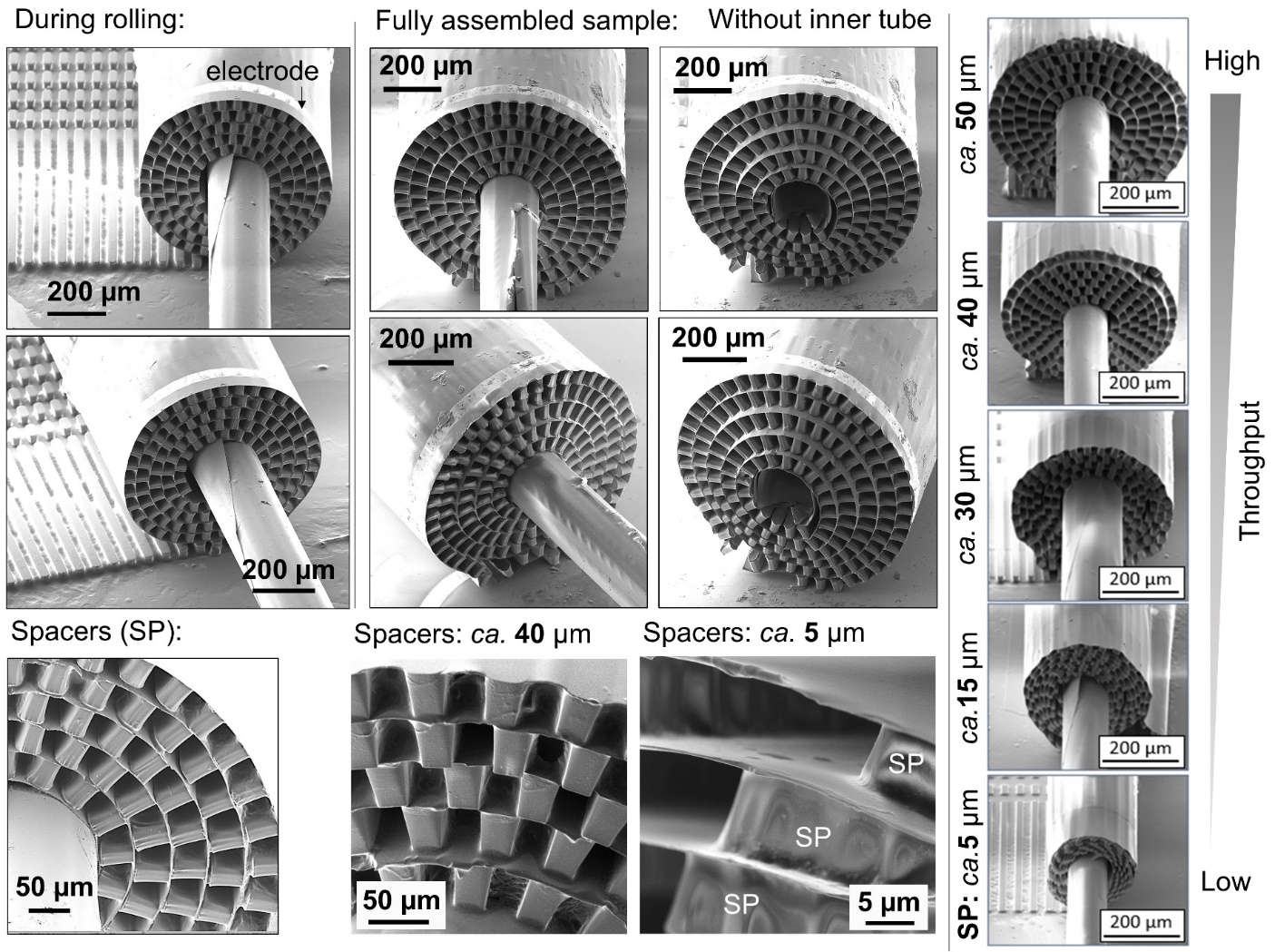


**Fig. S4** Scanning electron microscopy (SEM) images of self-assembled Swiss-roll coaxial platform with varying interwinding gaps. The top row shows the fabrication process, illustrating the rolling of a layered structure into a coaxial configuration (left), resulting in the fully assembled samples (centre). The fully assembled samples exhibit a consistent pattern of concentric rings around a central inner tube, with the interwinding gap (spacer) distance systematically varied (right). The bottom rows show higher magnification views of the spacers for samples with ca. 40 µm and ca. 5 µm gaps, respectively, highlighting the microstructural morphology. The right column presents a series of fully assembled samples with varying interwinding gaps: 5 µm (volume of outer tube is ~70 nL), 15 µm (volume of outer tube is ~200 nL), 30 µm (volume of outer tube is ~400 nL), 40 µm (volume of outer tube is ~550 nL), and 50 µm (volume of outer tube is ~700 nL), demonstrating a clear correlation between the spacer size and the resulting macroscopic structure. The gradient bar on the right indicates the relative throughput associated with each spacer size, with larger gaps yielding higher throughput.


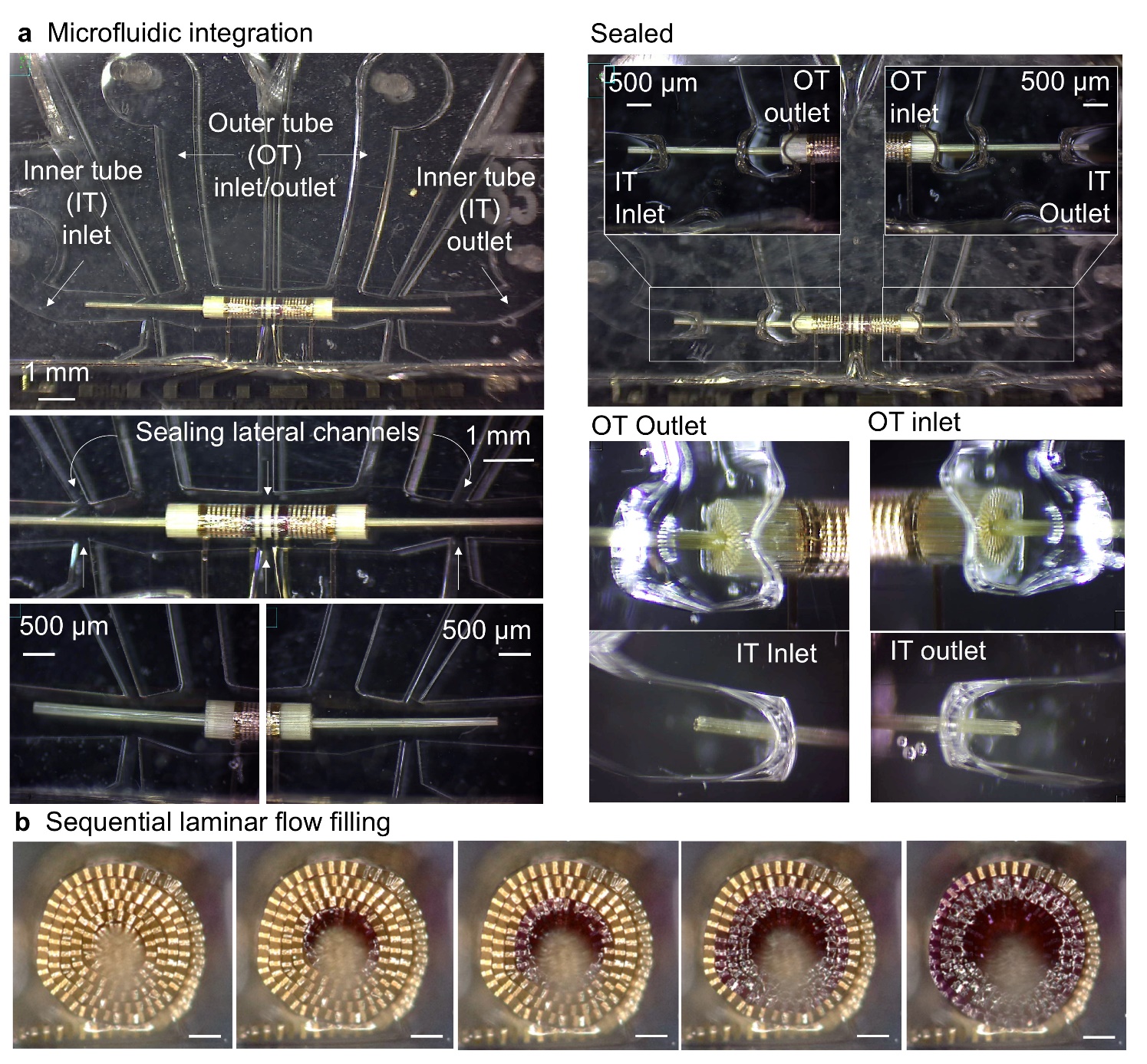


Fig. S5 Microfluidic integration and sealing of the 3D coaxial platform. **a** Sealing strategy. Optical micrographs showing the integration of the self-assembled coaxial structure within the PDMS device. The architecture is aligned in the main channel, and liquid PDMS is introduced through lateral channels to locally seal the structure, establishing isolated fluidic connections. **b** Programmable sequential filling. A series of optical micrographs demonstrating controlled, winding-by-winding fluid progression through the 3D architecture. The SU-8 spacer design creates discrete microfluidic channels within the windings, enabling precise control over the filled volume. This confirms the design provides addressable microfluidic access to the entire active surface area. Scale bars 50 µm.


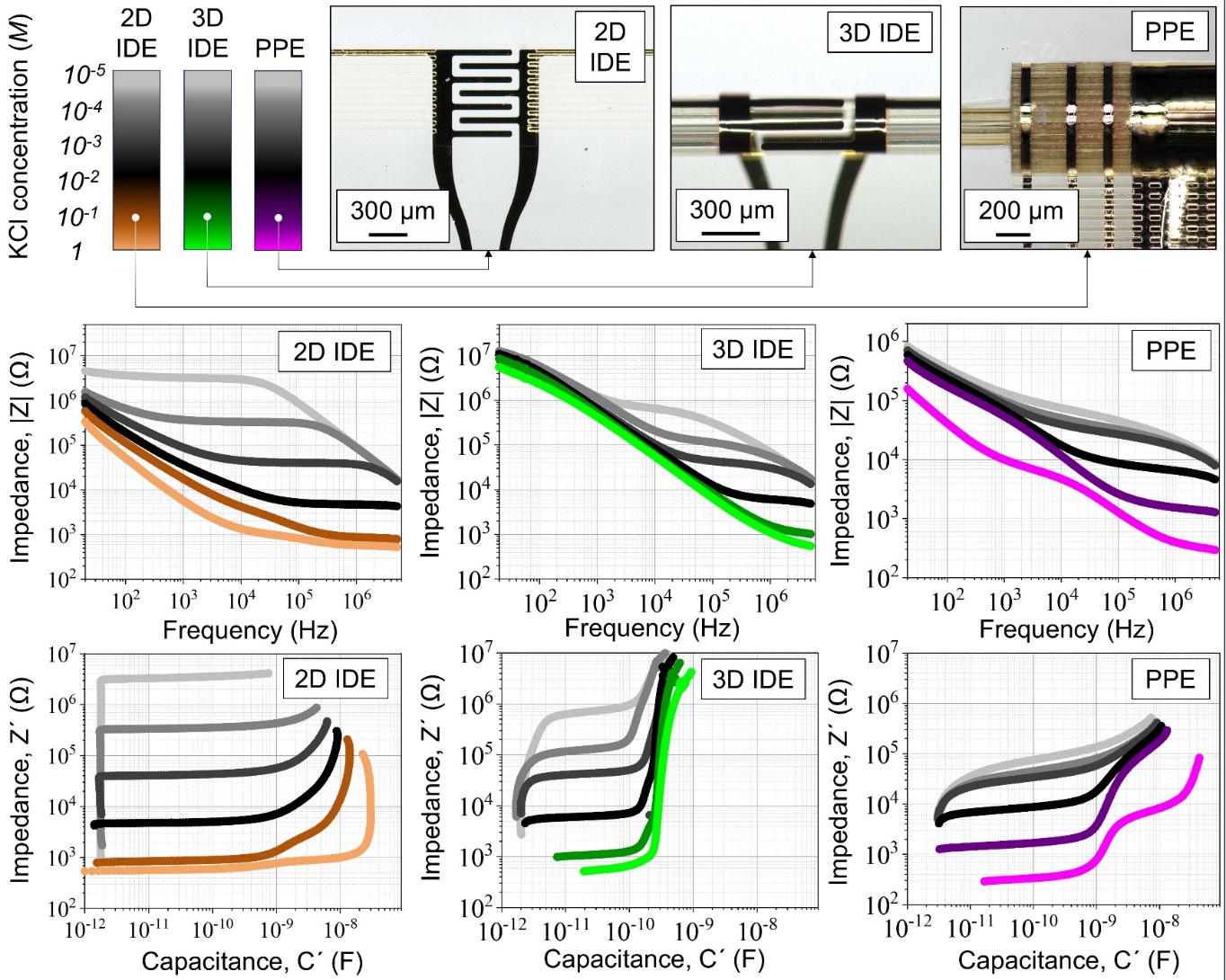


**Fig. S6** Geometry-dependent electrochemical performance of the integrated electrodes.
Bode plots (top row, impedance magnitude |Z| vs. frequency) and capacitance spectra (bottom row) directly compare the three electrode geometries - planar 2D IDEs, 3D coaxial IDEs, and PPEs - across KCl solutions (10^-5^ to 1 M). The data reveal a pronounced geometry effect: the 3D self-assembled structures (3D IDEs, PPEs) consistently outperform the 2D baseline, with PPEs providing the lowest impedance and most stable capacitance due to their large surface area, while 3D IDEs offer robust performance across all ionic strengths.


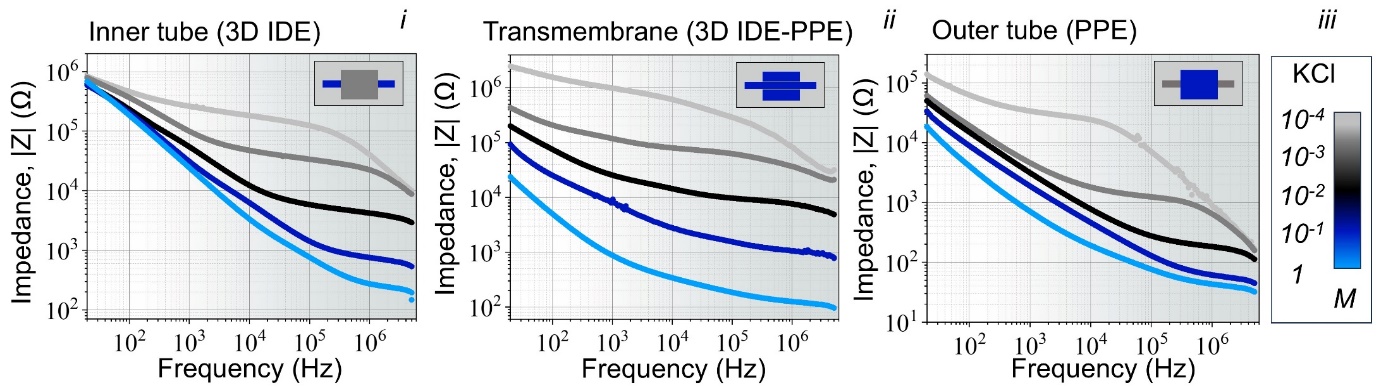


**Fig. S7** Bode plot analysis corresponding to Fig. 3c. Impedance magnitude (|Z|) versus frequency for the same measurements shown in the capacitance–impedance plots of Fig. 3c, featuring (*i*) 3D IDEs, (*ii*) 3D IDE-PPE pair, and (*iii*) PPEs across KCl solutions (10⁻^4^ to 1 M). These Bode plots provide complementary frequency-domain analysis, quantitatively showing that PPEs maintain the lowest impedance magnitude across most frequencies, while 3D IDEs offer stable performance over the broadest frequency range.


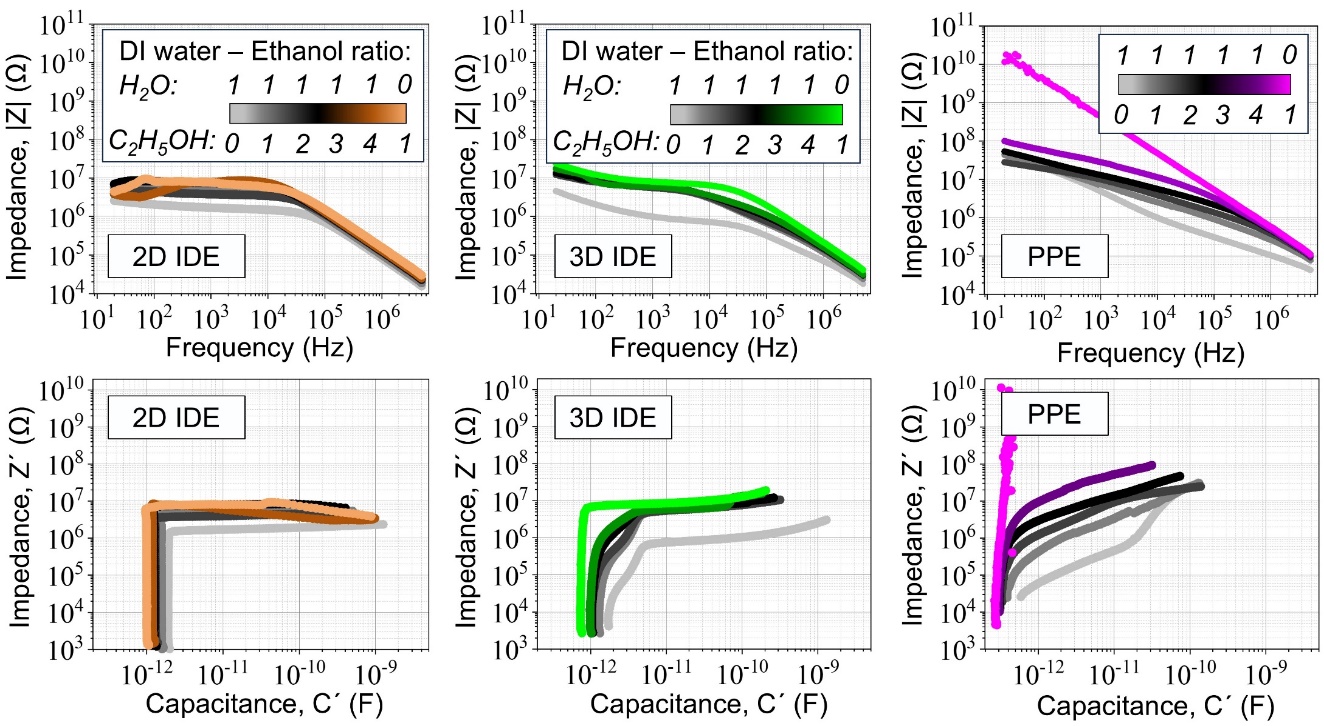


**Fig. S8** Dielectric sensitivity of electrode geometries. Bode plots (top row) and corresponding capacitance spectra (bottom row) for the 2D IDEs, 3D IDEs, and PPEs in ethanol–water mixtures of varying composition (pure water to pure ethanol). The data quantify the geometry-dependent dielectric response: PPEs act as highly sensitive dielectric sensors, with impedance varying by nearly three orders of magnitude, while the IDE geometries provide stable performance, making them robust for fluctuating environments. This demonstrates the capability to select electrode geometry based on the required application, whether sensing or stable operation.


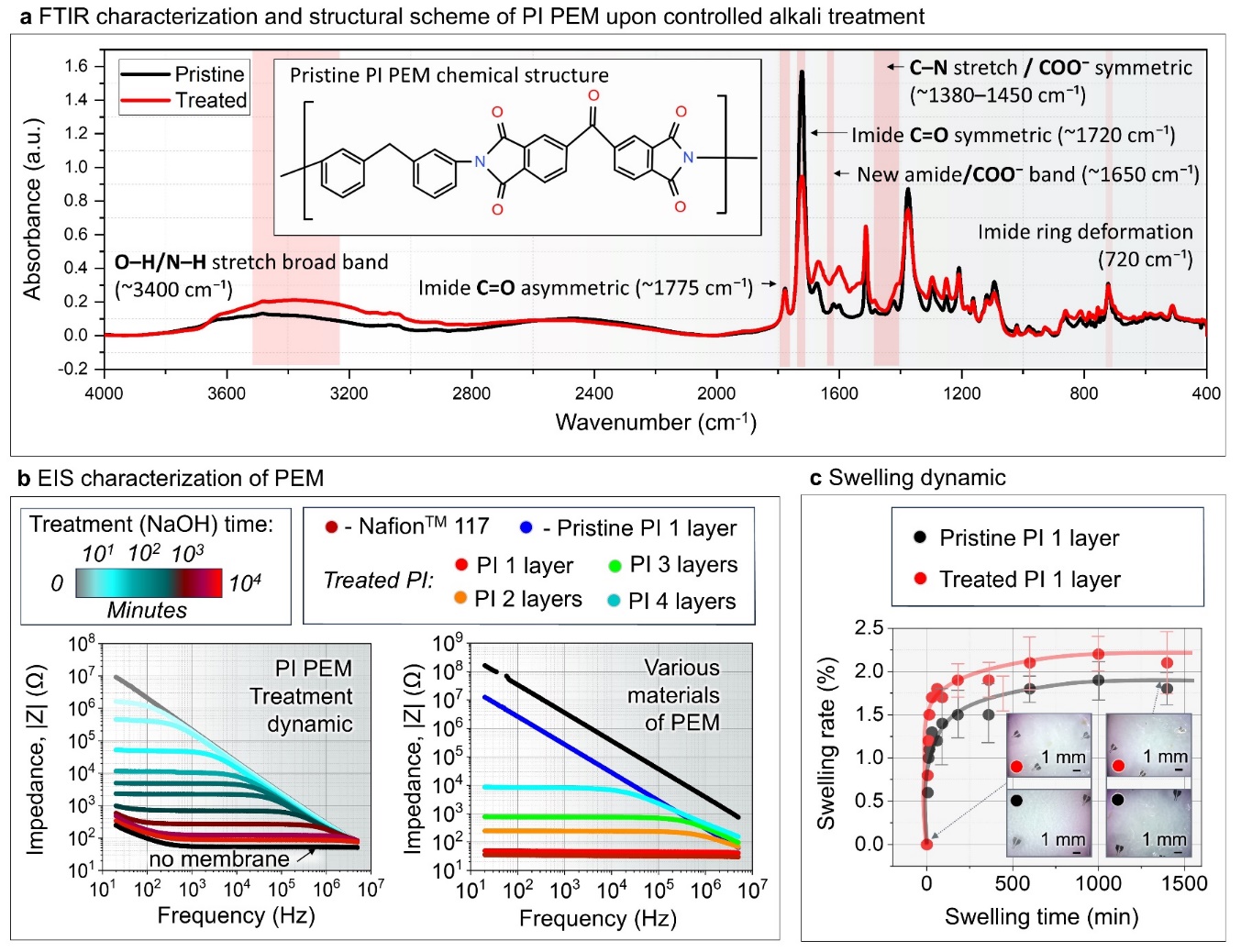


Fig. S9 Chemical and electrochemical evidence for PI PEM tunability. **a** FTIR spectra of pristine (black) and NaOH-treated (red) PI membranes. The treatment reduces characteristic imide peaks (asymmetric C=O stretch at ~1775 cm⁻¹, symmetric at ~1720 cm⁻¹) and introduces new bands (broad O–H/N–H at 3200–3600 cm⁻¹, amide C=O at ~1650 cm⁻¹, COO⁻ at ~1420 cm⁻¹), confirming the partial ring-opening into poly(amic acid) and carboxylate species that enhance hydrophilicity and proton conduction. **b** Electrochemical impedance spectroscopy (Bode plots, |Z| vs. frequency) provides the underlying data for the performance metrics in Fig. 4d. The plots show the systematic reduction of membrane resistance with NaOH treatment time (left, corresponding to Fig. 4d, ii) and benchmark the performance of the optimally treated PI against commercial standards (right, corresponding to Fig. 4d, iii). c Swelling dynamics of PI membranes. Time-resolved swelling of pristine (black) and NaOH-treated (red) PI membranes measured by optical tracking of metal markers over 24 h immersion in aqueous electrolyte. Pristine membranes exhibited 1.5–1.7% swelling, while treated membranes showed 2.1–2.3% swelling. The moderate expansion remains within the elastic limit of integrated gold electrodes, confirming mechanical compatibility.

**
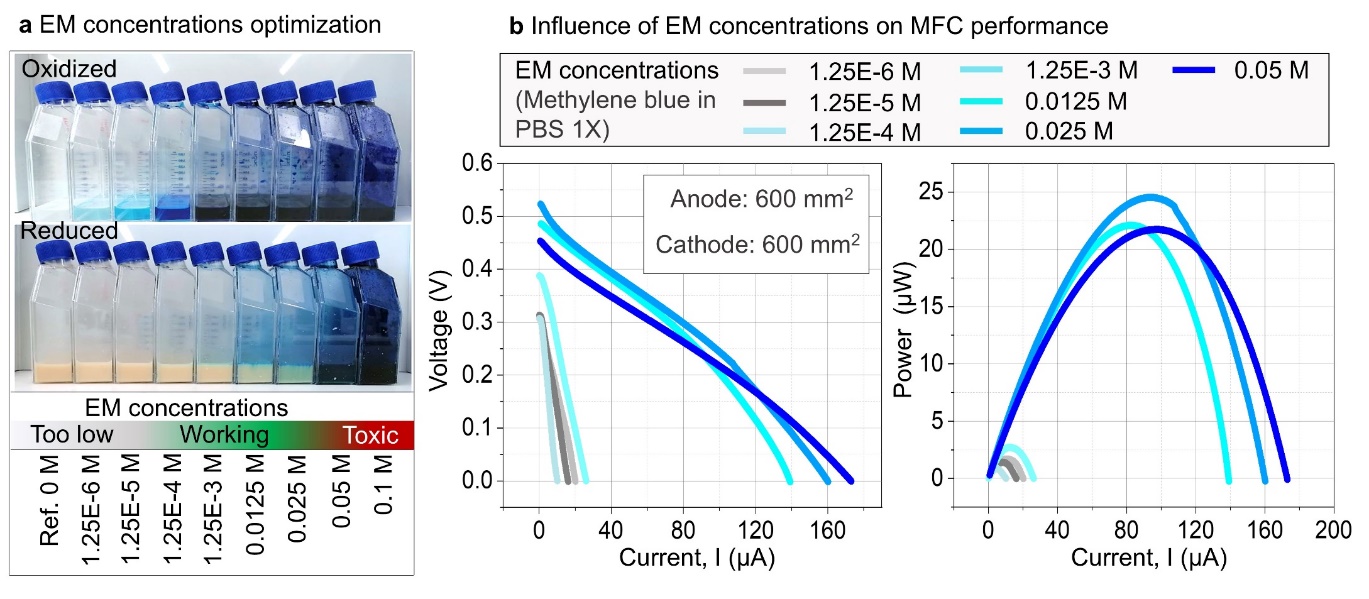
**

**Fig. S10** Influence of exogenous mediator concentration on system performance. **a** Photographs of methylene blue solutions, showing the oxidized (blue, top) and reduced (colorless, bottom) states across a concentration gradient. The visible color transition confirms active redox cycling within the operational range. **b** Polarization and power density curves quantifying the performance window. Optimal output occurs at mediator concentrations of 1.25 × 10⁻³ M to 0.025 M. Performance declines at lower concentrations due to insufficient electron transfer and at higher concentrations (≥0.05 M) due to cytotoxic effects.

**
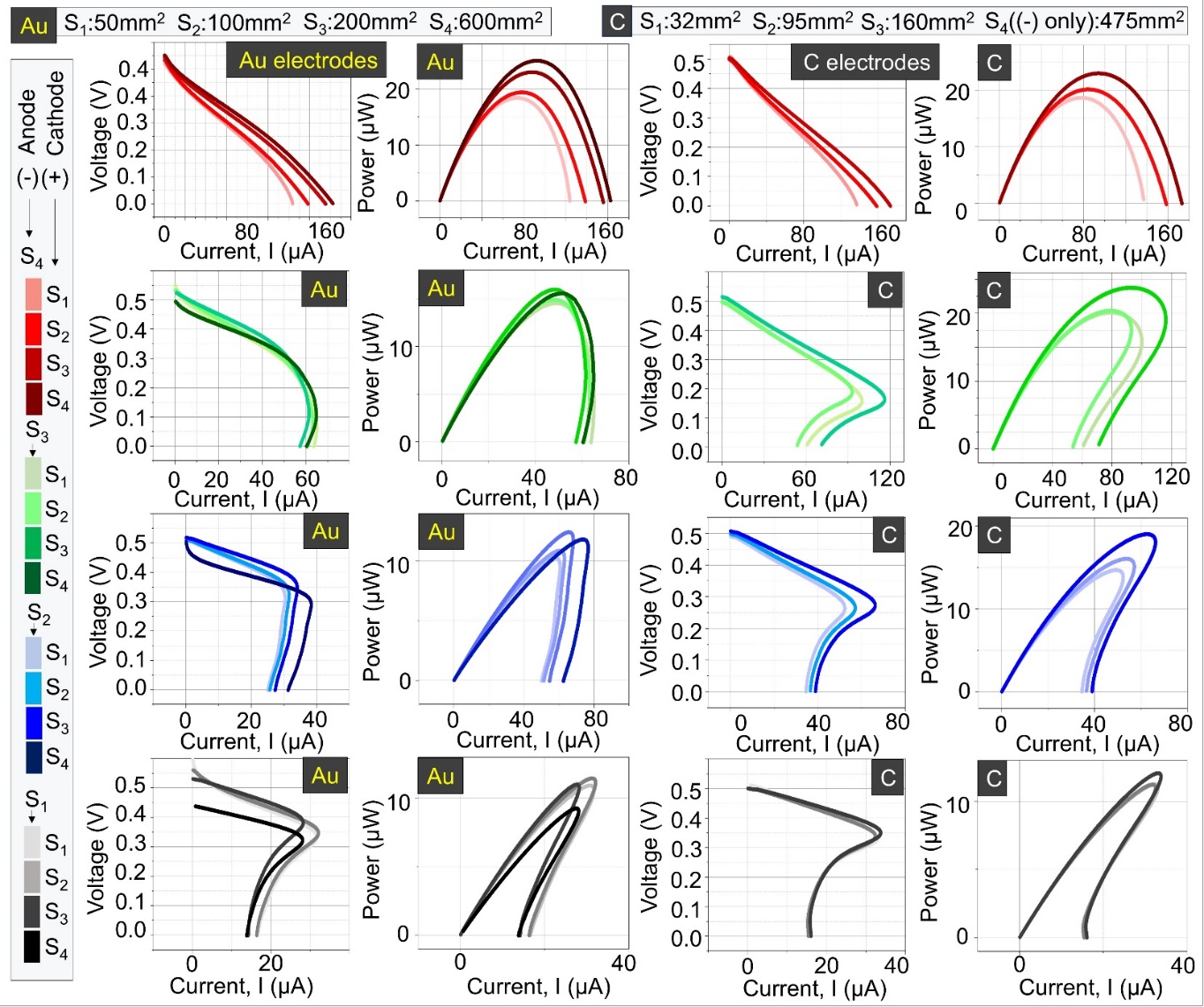
**

**Fig. S11** Electrode material and surface area optimization. Polarization and corresponding power density curves for gold (Au) and graphite (C) electrodes of increasing surface area (S1–S4). The data demonstrate that enlarging the anode surface area markedly improves current and power output, with Au electrodes consistently outperforming graphite. Cathode enlargement provided minimal performance gains. These results directly informed the selection of Au and the prioritization of anode area in the coaxial µMFC design.


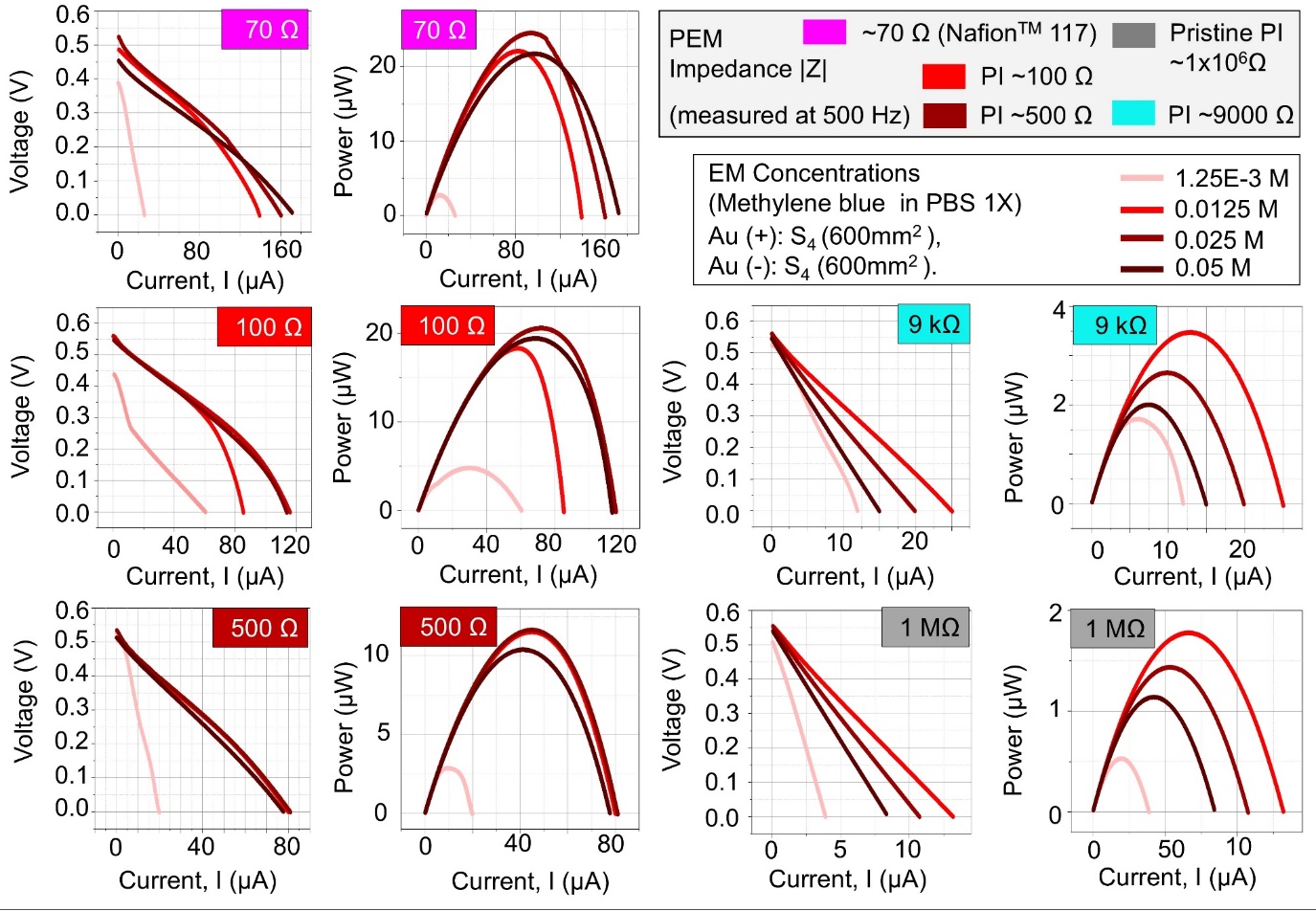


**Fig. S12** Independent optimization of PEM resistance and mediator concentration. Polarization and power density curves for PI proton-exchange membranes (PEMs) of varying resistance (70 Ω to 1 MΩ) across a range of methylene blue concentrations. The data demonstrate two independent design axes: (1) The optimal mediator concentration window (~0.0125–0.025 M) is consistent across all PEM resistances. (2) Within this optimal window, the absolute performance is exclusively dictated by PEM resistance, with low-resistance membranes (~70-250 Ω) enabling the highest power output.

**
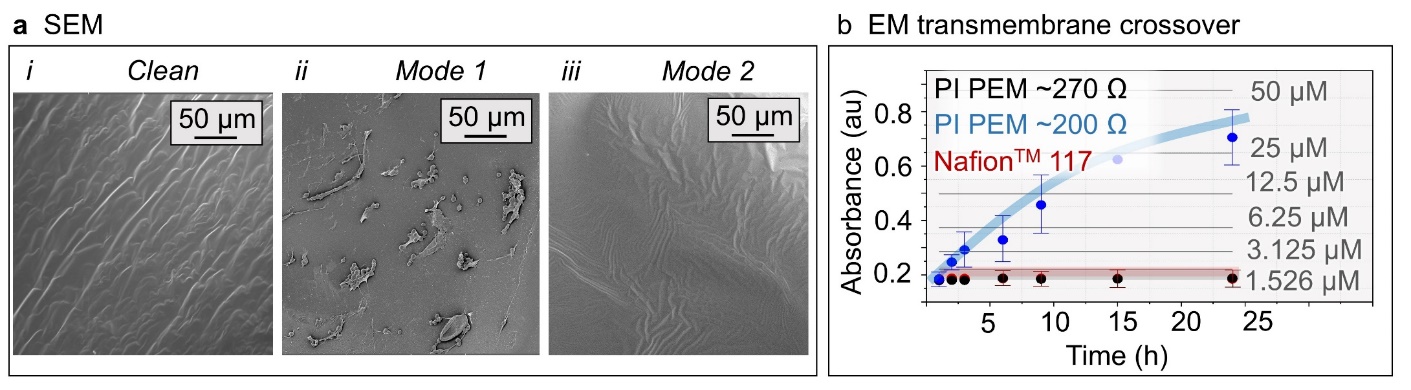
**

**Fig. S13** Biofouling and mediator crossover characterization. a SEM images of PEM surfaces under clean conditions (*i*) and after exposure to Mode 1 (*ii*) and Mode 2 (*iii*). Mode 1 shows extensive surface coverage and biomass aggregates, whereas Mode 2 retains the underlying membrane topography, indicating absence of biofilm formation. b EM crossover over 24 h for PI PEM (~200 Ω), PI PEM (~270 Ω), and Nafion™ 117; optimized PI PEM (~270 Ω) blocks mediator transport below detection limit.


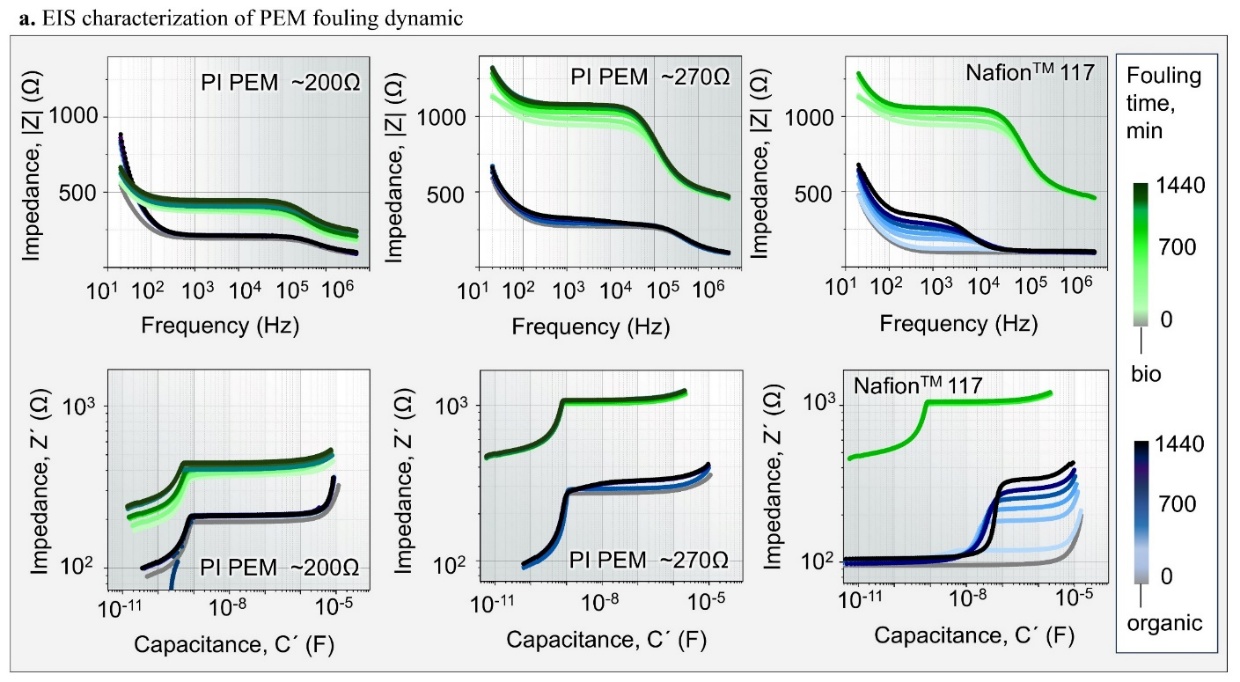


**Fig. S14** EIS characterization of PEMs. Bode plots (top row, impedance magnitude |Z| vs. frequency) and capacitance spectra (bottom row) directly compare the three PEMs: PI PEM ~200 Ω (left), PI PEM ~270 Ω (central) and Nafion™ 117 PEM.

**Supplementary Videos**

**Video S1** Independent and stable fluidic operation of inner and outer channels.

The video demonstrates the integrity of the integrated microfluidic platform, showing independent injection of orange (inner channel) and green (outer channel) fluid streams. Flow is pulsed in forward and reverse directions, confirming complete isolation between the two coaxial compartments with no observable mixing, as illustrated in the connection scheme in Fig. 3b. (Playback speed: 2x real-time)

**Video S2** Controlled fluidic transfer between inner and outer channels.

This video illustrates the designed fluidic routing capability. A red dye introduced into the inner tube transfers to the outer tube via an external interconnection, subsequently flowing in the opposite direction within the outer coaxial channel. This demonstrates programmable fluidic control within the sealed microsystem. (Playback speed: 2x real-time)

**Video S3** Independent filling of inner and outer channels in an open configuration.

Deionized water is shown independently filling the inner and outer tubes of the Swiss-roll structure, confirming the successful self-assembly of two distinct, continuous microfluidic channels. (Playback speed: 2x real-time)

**Video S4** Sequential and controlled filling of the interwinding channels.

In an open configuration, deionized water fills the interstitial channels between the windings in a sequential manner, as guided by the SU-8 spacer design (related to Supplementary Fig. S5b). This demonstrates controlled fluidic access to the entire 3D geometry and the potential for localized fluidic addressing. (Playback speed: 2x real-time)

**Video S5** Visualization of sequential interwinding filling with colored fluid.

A colored dye visually enhances the demonstration of the sequential filling process, clearly showing the controlled progression of fluid through the interwinding channels one by one, confirming efficient fluidic access to the coiled architecture. (Playback speed: 2x real-time)
